# Probing heavy metal binding to phycobiliproteins

**DOI:** 10.1111/febs.16396

**Published:** 2022-02-22

**Authors:** Jeddidiah Bellamy‐Carter, Jaspreet K. Sound, Aneika C. Leney

**Affiliations:** ^1^ 1724 School of Biosciences University of Birmingham UK

**Keywords:** heavy metal toxicity, light‐harvesting complex, native mass spectrometry, phycobiliproteins, protein‐metal interactions

## Abstract

Blue‐green algae, also known as cyanobacteria, contain some of the most efficient light‐harvesting complexes known. These large, colourful complexes consist of phycobiliproteins which are extremely valuable in the cosmetics, food, nutraceutical and pharmaceutical industries. Additionally, the colourful and fluorescent properties of phycobiliproteins can be modulated by metal ions, making them highly attractive as heavy metal sensors and heavy metal scavengers. Although the overall quenching ability metal ions have on phycobiliproteins is known, the mechanism of heavy metal binding to phycobiliproteins is not fully understood, limiting their widespread quantitative applications. Here, we show using high‐resolution native mass spectrometry that phycobiliprotein complexes bind metal ions in different manners. Through monitoring the binding equilibria and metal‐binding stoichiometry, we show in particular copper and silver to have drastic, yet different effects on phycobiliprotein structure, both copper and silver modulate the overall complex properties. Together, the data reveals the mechanisms by which metal ions can modulate phycobiliprotein properties which can be used as a basis for the future design of metal‐related phycobiliprotein applications.

## Graphical abstract

Native mass spectrometry reveals differential binding of metal ions to the colourful fluorescent phycobiliproteins from cyanobacteria. The results presented in this study have a broad impact on the use of phycobiliproteins in biosensors and in predicting the response of cyanobacteria to heavy‐metal contaminants within the environment.

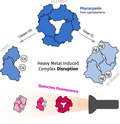

**Abbreviations**
APC: allophycocyanin
MS: mass spectrometry
PC: phycocyanin
PCB: phycocyanobilin

## Introduction

Phycobiliproteins, found in microalgae, are among the most brilliantly colourful and fluorescent proteins in nature. They are high‐value natural products in the food, cosmetic, nutraceutical and pharmaceutical industries [1]. Additionally, their extremely high extinction coefficients and quantum yields [2] make phycobiliproteins extremely attractive as fluorescent dyes [2, 3]. The applications of phycobiliproteins are continuously expanding. Phycobiliproteins have more recently been exploited due to their ability to bind metal ions. Phycocyanin (PC), the most abundant phycobiliprotein in blue‐green microalgae, binds heavy metal ions and in doing so quenches its fluorescence. As such, PC has been proposed as a good biosensor for heavy metals, especially mercury (Hg^2+^) for which it has a very high affinity [4, 5, 6, 7]. Related to this application, PC has a number of therapeutic abilities as a heavy metal scavenger [8, 9, 10, 11, 12, 13]. Phycobiliproteins have also shown promise in the development of silver nanoparticles whereby encapsulation by PC has been shown to reduce their toxicity [14]. Despite these numerous applications, however, the fundamental mechanisms behind how phycobiliproteins bind metal ions remain to be deciphered.

**Fig. 1:**
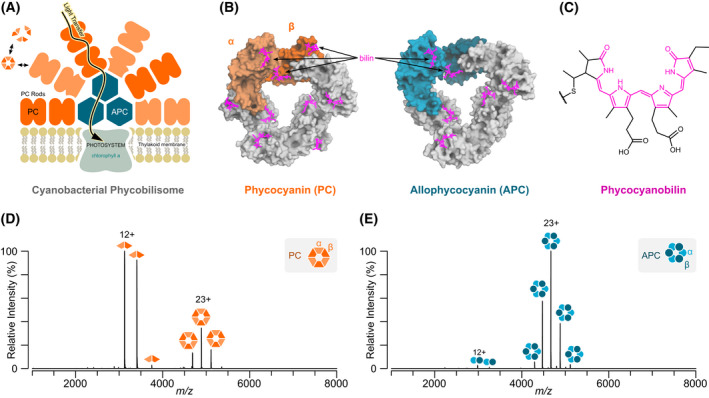
Cyanobacterial phycobiliproteins. (A) Schematic of a cyanobacterial phycobilisome showing the role of PC and APC in light transfer along with their location within the complex. (B) 3D surface representations of PC and APC hexamers, αβ dimers are coloured and PCB groups are highlighted in pink. Structures were generated in pymol (The PyMOL Molecular Graphics System, Version 2.4, Schrödinger LLC, New York, NY, USA) from PDB entries 1GH0 and 3DBJ, respectively. (C) Chemical structure of PCB, the sole bilin found on PC and APC, with fluorophore highlighted. (D) Native mass spectrum of purified PC and (E) APC (each at 1 μm hexamer).

Phycobiliproteins function in microalgae as a crucial part of photosynthesis, facilitating a cascade of light energy towards the photosystem and chlorophyll as part of a mega‐complex known as the phycobilisome (Fig. 1A) [15, 16, 17]. In blue‐green algae, the most abundant of these phycobiliproteins are the blue‐coloured PC (λ_max_ = 620 nm) [18] and cyan‐coloured allophycocyanin (APC; λ_max_ = 650 nm) [8]. All phycobiliproteins are built from conserved α and β subunits, each with bilins, which form strong heterodimers. These αβ dimers readily form higher‐order hexamer (α_3_β_3_; Fig. 1B) and dodecamer (α_6_β_6_) discs which stack into rods with the aid of linker proteins to form the intact phycobilisome. The colour and fluorescent properties of phycobiliproteins are imbued by conserved bilin prosthetic groups, predominantly phycocyanobilin (PCB; Fig. 1C) and phycoerythrobilin whose orientation within the hexameric sub‐complexes within the phycobilisome is essential for its effective colour and high fluorescence. It has been proposed that metal ions bind to the bilins and in doing so modulate phycobiliprotein fluorescence; this may be one of the mechanisms by which heavy metals lead to cellular toxicity [5, 13, 19]. Spectroscopic studies have shown PC to be quenched by a number of metals, in particular Cu^2+^ and Hg^2+^, with other metals including Ag^+^ having a moderate effect on fluorescence [4, 6]. Molecular docking studies of selected metal ions with PC suggest diverse binding sites, typically involving acidic sidechains [7]. There are, however, unanswered questions regarding the stoichiometry of metal binding and the mechanism by which metal‐binding quenches fluorescence within the phycobiliprotein protein complexes. Indeed, the binding mechanism may be different for different metal cations although the overall change in fluorescence displays similar properties. Intriguingly, in contrast to PC, APC is curiously unexplored regarding metal binding, yet it is structurally similar and harbours the same bilin chromophores.

Here, we use native mass spectrometry (MS) to probe the mechanistic details behind how the phycobiliproteins, PC and APC, bind metal cations. Native MS allows protein and protein complexes to be analysed in their native‐like state, preserving stoichiometry and binding equilibria between proteins and protein complexes, enabling us to determine how these are altered upon metal binding [20, 21, 22, 23, 24]. Phycobiliproteins have previously been successfully investigated by native MS showing that their native structure can be preserved [25, 26, 27]. Four metal cations, Ag^+^, Fe^2+/3+^, Zn^2+^ and Cu^2+^, were selected based on their differential ability to quench PC fluorescence [4, 6] and their binding to PC and APC probed using native MS. With both PC and APC, no/little binding was observed with Fe^2+/3+^ and Zn^2+^, consistent with their minimal effect on phycobiliprotein fluorescence. However, we observe large structural changes to PC upon the addition of Cu^2+^ and Ag^+^, highlighting how these metals can differentially regulate PC fluorescence. Overall, we reveal the molecular details behind heavy metal binding to phycobiliproteins; the information from which will be essential for the further development of novel biotechnological applications of phycobiliproteins.

## Results

### Allophycocyanin binds differentially to metal cations

To demonstrate the applicability of native MS to monitor phycobiliproteins, APC and PC were first taken and analysed using native MS (Fig. 1D,E). Narrow charge state distributions were observed corresponding to both the APC and PC (αβ)_3_ hexamers showing that native MS can capture the native states of these fluorescent complexes. Interestingly, the αβ PC dimer is also observed at lower *m/z* regions suggesting that PC is in dynamic equilibrium between its dimeric and hexameric states. This is in sharp contrast to APC which is almost entirely hexamer at concentrations that give predominantly dimer for PC (Fig. 1).

**Fig. 2:**
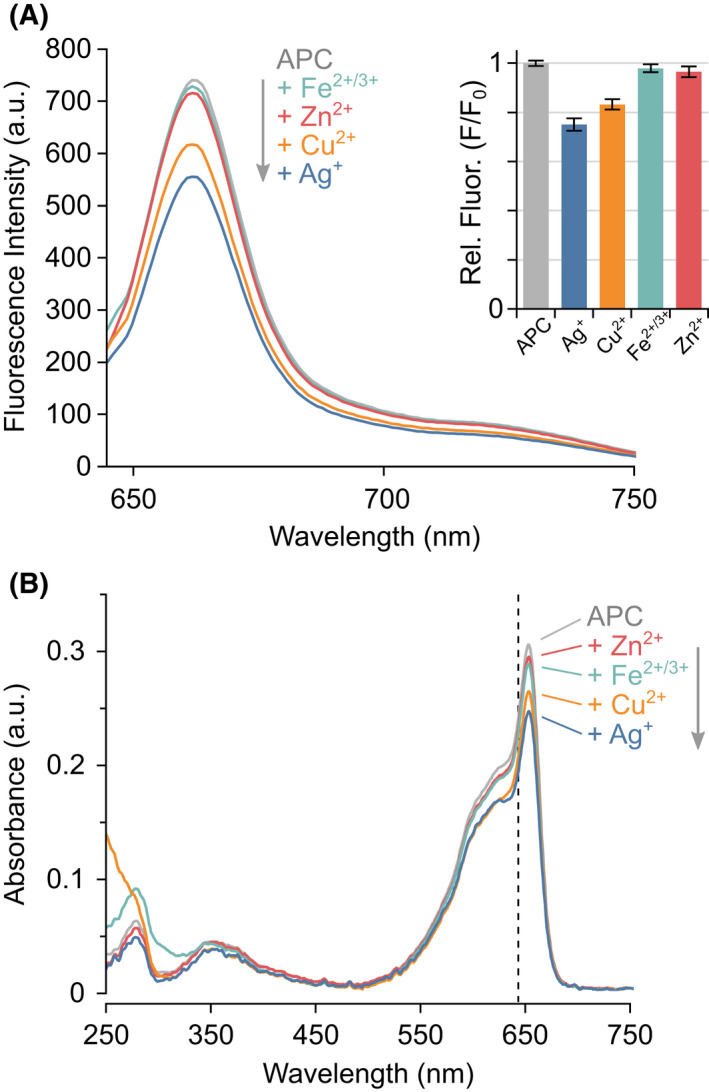
Spectrophotometric quenching of purified APC by metal ions. (A) Fluorescence emission spectra (excited at 645 nm, marked as a dashed line in (B). The inserted bar graph shows relative fluorescence at 663 nm (error bars indicate ± SD, *n* = 5). (B) Visible light absorbance spectra of purified APC after incubation in the absence (grey) or presence of metal ions: Ag^+^ (blue), Cu^2+^ (orange), Fe^2+/3+^ (teal) or Zn^2+^ (red).

**Fig. 3:**
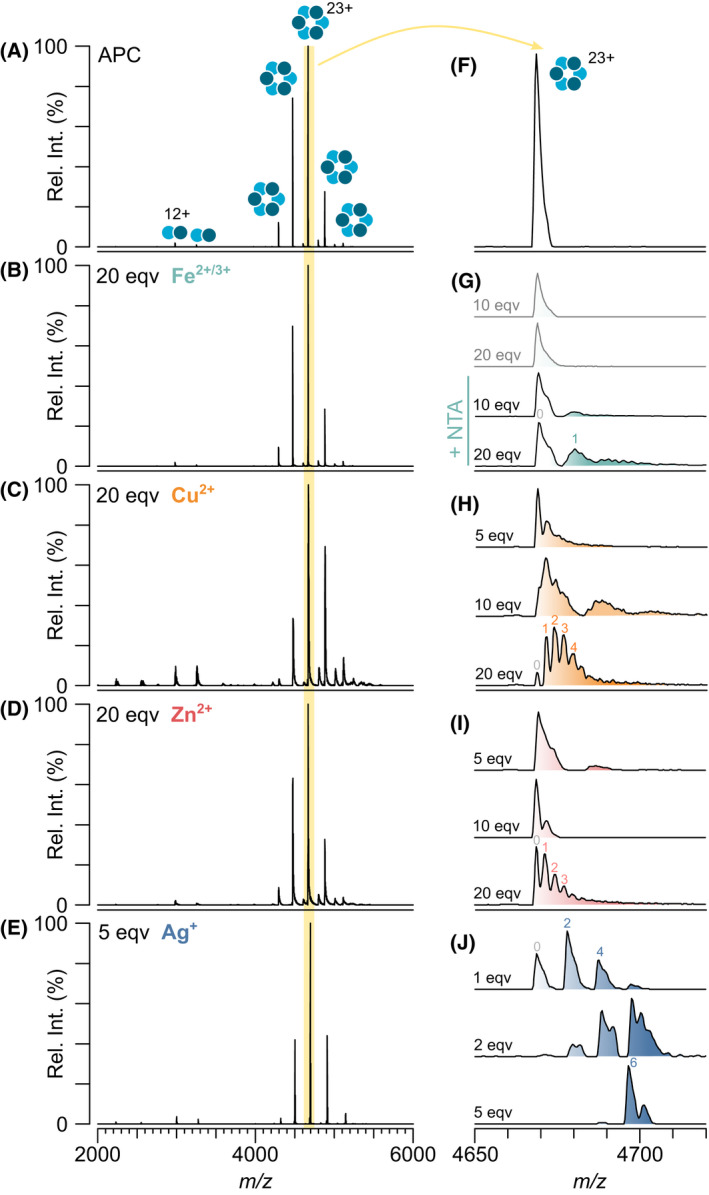
Native mass spectra show Ag^+^ and Cu^2+^ bind purified APC with high affinity. Control with no metals (A) and APC upon addition of Fe^2+/3+^ (B), Cu^2+^ (C), Zn^2+^ (D) or Ag^+^ (E) with (F–J) corresponding to the same spectra with the number of metal ions bound to the 23+ charge states highlighted. Panel (G) shows the effect including nitrilotriacetic acid (NTA) chelator on Fe binding. Intensity units are shown as relative intensity (rel. int.). Shoulder peaks for the silver bound APC hexamer correspond to addition of ~ 50–70 Da which is a combination of NH_4_^+^, Na^+^, K^+^ and CO_3_^‐^ adducts. At 5 equivalents of Ag^+^, a small amount of APC + 7 Ag^+^ is observed.

Next, metal binding to APC was probed using native MS. APC has been somewhat overlooked with regard to metal‐induced fluorescence quenching with the most insightful studies not looking at APC in isolation.[28, 29] However, due to the structural similarities between APC and PC and the identical bilins they harbour, one might expect them to show similar effects. Purified APC was mixed separately with four metal ions, Ag^+^, Cu^2+^, Fe^2+/3+^ and Zn^2+^, and their binding to APC was monitored by absorbance (Fig. 2A) and fluorescence (Fig. 2B) spectroscopy in combination with native MS (Fig. 3). Little change in fluorescence was observed upon the addition of Fe^2+/3+^ and Zn^2+^ with more quenching observed by Cu^2+^ and Ag^+^ (Fig. 2A). The decrease in fluorescence quenching upon the addition of different metal ions correlates with their binding to APC as determined by native MS: Ag^+^ > Cu^2+^ > Zn^2+^ > Fe^2+/3+^ (Fig. 3). No or very little binding was observed with iron even with the addition of a chelating agent, nitrilotriacetic acid (NTA), to stabilise the ion (Fig. 3B,G). Some zinc binding to APC was observed but only when zinc was present in a 20‐fold excess (Fig. 3D,I). Copper bound with a slightly higher affinity, whereby 1 Cu^2+^ was found to add sequentially to the APC hexamer (Fig. 3C,H). Silver showed an even greater affinity for APC, amassing six Ag^+^ bound to the hexamer at 5 bilin equivalents (Fig. 3e,J). Oddly, the binding of silver to APC occurs in 2 Ag^+^ sequential steps hinting that its mechanism of binding is different to that of Cu^2+^ and Zn^2+^. The native MS data shows the equilibrium between the oligomeric states of APC does not change upon addition of metal ions on a short timescale (Fig. 3); however, prolonged exposure to Cu^2+^ leads to significant dissociation towards the dimer. The absorbance spectra for APC with these metal ions show little shift in λ_max_ but some decrease in total absorbance (Fig. 2B) suggesting that only moderate structural perturbations occur upon metal binding to APC.

### Major perturbation of phycocyanin structure upon binding of Cu^2+^ and Ag^+^

**Fig. 4:**
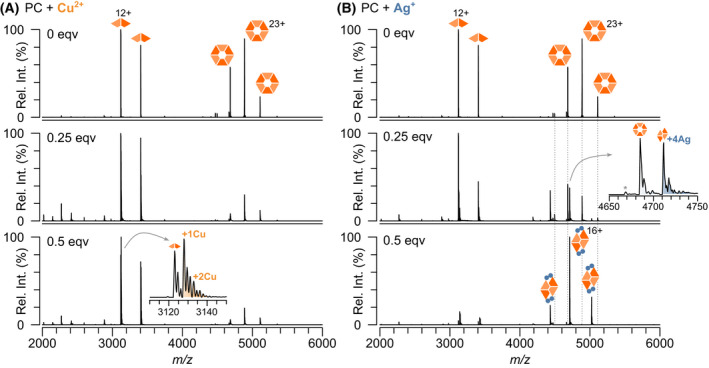
Native mass spectra show differential binding of Ag^+^ and Cu^2+^ to purified PC. PC (24 μm bilin) upon addition of Cu^2+^ (A) and Ag^+^ (B) at 0, 0.25 and 0.5 bilin equivalents. Copper ions cause the PC hexamer to dissociate into a dimer. Silver ions cause the PC hexamer and dimer to shift to a tetramer conformation. Dashed lines indicate the position of the PC hexamer ions that disappear upon silver binding.

**Fig. 5:**
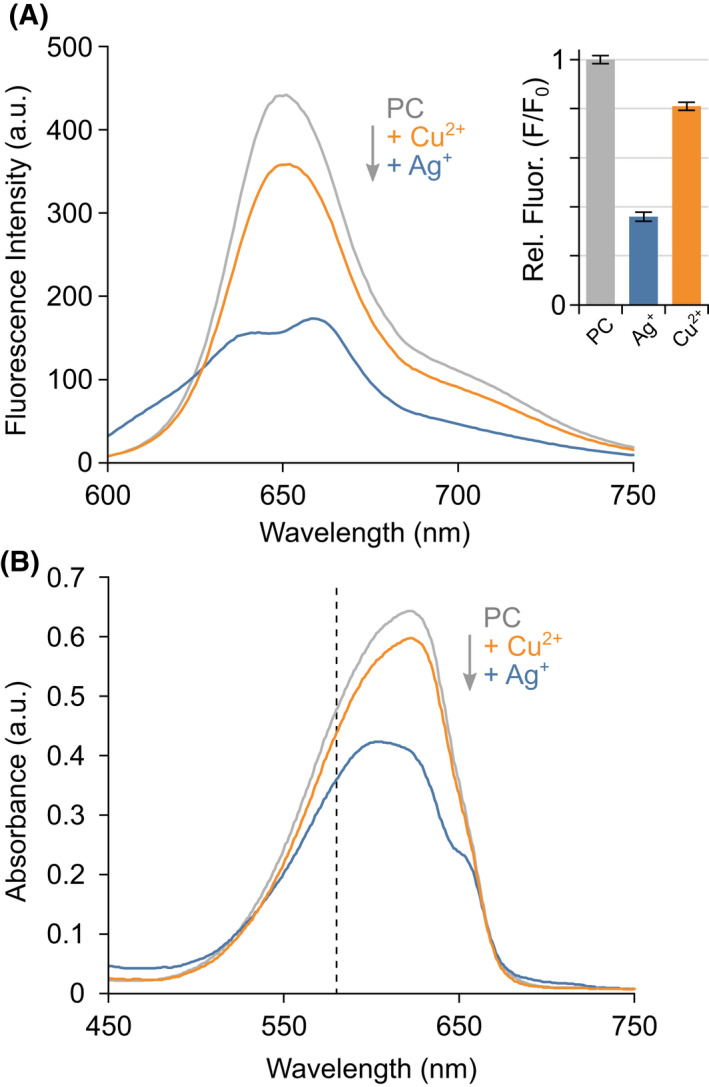
Spectrophotometric quenching of purified PC by metal ions. (A) Fluorescence emission spectra (excited at 580 nm, marked as dashed line in B), inset bar graph shows relative fluorescence at 652 nm (error bars indicate ± SD, *n* = 5). (B) Visible light absorbance spectra of PC after incubation in the absence (grey) or presence of metal ions: Ag^+^ (blue) or Cu^2+^ (orange).

Next, the effect of metal ion binding to PC was probed using native MS. In sharp contrast to APC, large spectral changes were observed upon the addition of copper and silver, even at a lower excess (0.5‐fold excess over the normalised bilin concentration; Fig. 4A). For Cu^2+^, the PC dimer‐hexamer equilibrium is shifted with almost only dimer observed after 45 min of incubation. Despite this shift in equilibrium, the stoichiometry of Cu^2+^ binding to the dimer and hexamer ions shows a considerable proportion of unbound PC, suggesting that Cu^2+^ could disrupt hexamer assembly. As with APC, the Cu^2+^ binds in one Cu increments. The effect of silver is most intriguing. Upon addition of small amounts of silver, again the dimer‐hexamer equilibrium is perturbed, but this time an (αβ)_2_ tetramer predominates (Fig. 4B); an oligomeric state not previously detected for PC. It is noteworthy that the charge state distribution observed for the tetramer overlaps the hexamer—if the oligomers had similar charge density (as would be expected for globular proteins), the tetramer would be expected to be at much lower *m/z* than the hexamer. This suggests that the tetramer has a much lower solvent‐accessible surface area for its mass than the hexamer—in other words, it is more compact and near‐spherical than an open ‘partial‐ring’ tetramer might be. This tetramer species initiates with four Ag^+^ bound, with no apparent lower intermediate. Although the apparent affinity of these two metals for PC and APC follow the same order (Ag^+^ > Cu^2+^), they both exhibit much greater affinity for PC and, crucially, the effects on oligomerisation could not be more different. These structural changes correlate with an overall decrease in absorbance (Fig. 5A) and fluorescence (Fig. 5B) of PC. The binding of Ag^+^ and structural arrangements upon Ag^+^ binding to PC have a more dramatic effect on fluorescence compared with Cu^2+^ suggesting that tetrameric PC is not sufficient for light transfer. Recently, a PC from a thermophilic cyanobacterium has been observed to temporarily occupy a non‐conventional hexadecameric structure which is proposed to form via an intermediate tetrameric state, consisting of two stacked αβ dimers [30]. Thus, it is possible a similar structure is being forced upon PC from *Arthrospira maxima* under the influence of silver.

### Phycocyanin outcompetes allophycocyanin in its metal‐binding characteristics

**Fig. 6:**
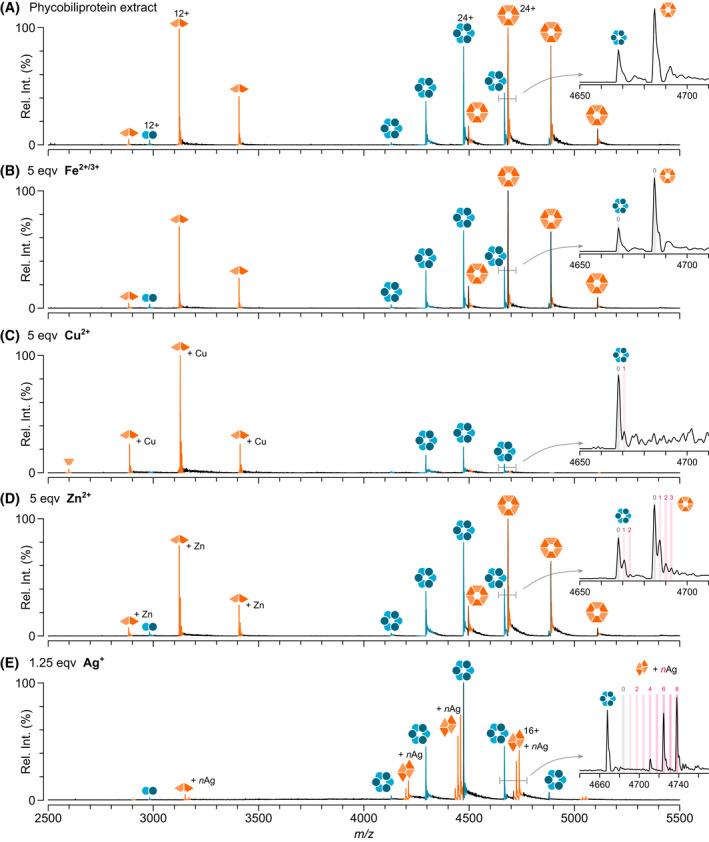
Native mass spectra reveal differential binding of metal ions to phycobiliproteins. Phycobiliproteins extracted from *Arthrospira maxima* (1475/9) (24 μm normalised bilin concentration) without metal ions (A) and upon addition of 120 μm Fe^2+/3+^ (B), 120 μm Cu^2+^ (C), 120 μm Zn^2+^ (D) and 30 μm Ag^+^ (E). The PC and APC complexes are shown in orange and blue, respectively.

Finally, we probed the effect of metal binding to a mixture of phycobiliproteins extracted from the same species, *A. maxima* (CCAP 1475/9). Consistent with APC and PC analysed individually, when present together, APC was predominantly hexameric whereas PC was present in both its dimeric and hexameric forms (Fig. 6A). Due to the high resolution of the Orbitrap mass analyser, the stoichiometry of metal binding to APC and PC could be discerned from the same spectra (Fig. 6 inserts). Despite the natural differences in abundance of the phycobiliproteins, APC and PC bound Fe^2+/3+^ and Zn^2+^ in a similar manner to purified APC and PC upon addition of a 5‐fold excess of Fe^2+/3+^ and Zn^2+^, showing no change in the oligomeric status of PC or APC (Fig. 6B,D). Copper again had a minimal effect on the APC hexamer, but had a drastic effect with PC, whereby no PC hexamer remained (Fig. 6C). Silver also showed minimal binding to the APC hexamer with Ag^+^ observed predominantly bound to the newly formed PC tetramer (Fig. 6E). Again, multiples of two Ag^+^ ions were seen bound to PC with 8 Ag^+^ bound to the PC tetramer being the dominant species observed.

Overall, the binding of metals observed by native MS correlates with changes in the absorbance and fluorescent properties of the PC and APC in solution (Figs 2 and 5). This points away from non‐specific binding that can sometimes occur during the electrospray process during native MS [31, 32]. Importantly also in our case, PC without the addition of metal ions is observed as a dimer and hexamer with no trimer, tetramer, pentamer, heptamer and octamer observed (Fig. 1D) thus no non‐specific protein‐protein interactions are evident. Additionally, differential binding between metal ions and APC is observed at the same metal concentrations (Fig. 3), silver binds in multiples of two (Figs 3J and 6E), and silver binds preferentially to PC over APC when added to a mixture of both phycobiliproteins. These factors all indicate that these interactions we observe are occurring in solution and are of exciting biological relevance.

Taken together, the data on the phycobiliprotein extract shows that cation binding and structural rearrangements predominantly occur on PC and thus the mechanism behind heavy metal toxicity in algae likely centres around rod disruption or the prevention of rod assembly within the phycobilisome photosynthetic complex.

## Discussion

Phycobiliproteins have tremendous light absorbing and light transfer properties making them extremely attractive as dyes in the food and cosmetic industry. The binding of metal ions to phycobiliproteins can modulate these exciting properties which has led to the application of phycobiliproteins as heavy metal sensors and metal scavengers. However, the binding of metal ions to phycobiliproteins is not always advantageous. When phycobilisome‐containing cyanobacteria are exposed to water sources contaminated with heavy metal pollutants, the cyanobacteria typically die, leading to ecosystem imbalance. Increased fundamental understanding of how phycobiliproteins bind metal ions is needed. Thus, we set out to probe metal binding to phycobiliproteins using a combination of UV‐vis spectroscopy, fluorescence spectroscopy and native MS. We posed two questions: firstly, do heavy metals bind to PC and APC? And secondly, what might the mechanism of metal binding be? The answers to these questions are largely summarised in Fig. 6. PC and APC bind metal cations differently. Minimal binding of zinc and iron was observed to both APC and PC (Figs 3 and 6). However, a median of 2 copper ions was bound to the APC hexamer when Cu^2+^ was in a 20‐fold excess (Fig. 3C), and a median of 6 silver ions when Ag^+^ was in a 5‐fold excess (Fig. 3E). In sharp contrast, the PC hexamer completely dissociated into its dimeric counterparts upon addition of copper (Fig. 6C) and with only a small amount of silver the PC hexamer completely rearranged into a tetrameric complex (Fig. 6D).

The difference in copper and silver binding to phycobiliproteins is intriguing. Both PC and APC from cyanobacterial species utilise only a single phycobilin, namely PCB, the location of which is conserved across the entire superfamily. The APC hexamer bound six Ag^+^ which is consistent with its number of PCB chromophores (Fig. 1B). However, PC harbours three PCB moieties for each αβ dimer (1 on alpha and 2 on beta), and thus if the metal ions bound only to PCB, one would expect nine Ag^+^ bound to PC, but this is not the case. Moreover, eight Ag^+^ were observed bound to the re‐arranged PC tetramer that contains only six PCBs in total. Thus, the bilins may not be the only sites of metal binding. Indeed, silver can also bind amino acids such as Cys, Met, His, Lys, Arg and Trp [33] all of which could influence the PC inter‐dimer interfaces within the hexamer. Phycobiliproteins are natural fluorescence transfer systems, where the proximity of the bilins will determine the efficiency of light transfer; in the case of PC and APC, the most proximal PCBs occur at the inter‐dimer interface of the hexamer—thus disrupting this interaction leads to a reduction in fluorescence. Given that Cu^2+^ leads to a shift towards the dimer, it is likely that the Cu^2+^ coordinates to amino acids and bilins at the inter‐dimer interface, slowing the assembly of the hexamer. The formation of a compact tetramer by Ag would suggest that the Ag binds to residues different to Cu^2+^, having a greater impact on phycobiliprotein fluorescence. The difference in binding stoichiometries between Cu^2+^ and Ag^+^ is also striking. Cu^2+^ binds sequentially with one addition of Cu^2+^ to all complexes; however, Ag^+^ binds in multiples of two. Another explanation of the observed 2Ag binding is that aqueous silver can readily form neutral‐charge Ag_2_ nanoclusters, which can also bind amino acids [34]. The structure of phycobiliproteins, particularly PC, may, by chance, selectively bind these nanoclusters. Simultaneous binding of two silver ions has been hinted to occur previously by Sun and co‐workers, who saw two silver ions coordinating to one thiolate in the crystal structure of malate dehydrogenase [33]. However, whether a similar binding mechanism occurs in our case remains to be deciphered and will be the subject of further work. Indeed, other native MS studies involving Ag^+^ binding to insulin and metallothionein‐2A have shown that Ag^+^ readily binds in the expected single Ag^+^ stoichiometries [35, 36], suggesting this is not a native MS‐specific phenomenon.

The native MS data correlated nicely with UV‐vis spectroscopy and fluorescence spectroscopy data for metal binding to APC and PC. APC showed little change in the hexameric complex consistent with minimal changes in APC fluorescence, and the structural re‐arrangements upon adding copper and silver to PC correlated with a large decrease in PC fluorescence. Previous studies on PC fluorescence have used PC concentrations that are below the *K*_d_ of the PC hexamer and thus report on how metal ions influence the PC dimer. Here, the absorbance and fluorescence spectroscopy were performed under conditions similar to native MS experiments, i.e., on the more functionally relevant PC hexamer. In contrast to literature spectra, [4, 6] Ag^+^ now heavily quenches the fluorescence of PC (Fig. 5a), showing how deficient tetrameric PC is for light transfer.

## Conclusions

Overall, we have shown that both PC and APC bind heavy metal ions differentially, with the former having higher affinity for them. In particular, we have shown that both copper and silver result in a drastic change in the native oligomerisation of PC. We also show the surprising revelation that silver quenches PC fluorescence through the formation of an unnatural tetrameric conformation. These observations of facile PC‐reassembly effected by heavy metal ions explains the cytotoxic effects of heavy metal ions on algae [19] since the re‐arrangement of PC upon metal binding will alter the dynamics of phycobilisome assembly that is essential for phycobilisome function. Indeed, it is not just that these metals reduce the efficiency of light transfer through the phycobilisome; they also inhibit formation of, and essentially unravel, the phycobilisome—a phenomenon that is detrimental for cyanobacterial photosynthesis and thus their survival. Our findings help clarify the use of PC as a heavy metal biosensor, paving the way for a more quantitative approach to be employed. Moreover, we show that purified PC is extremely effective for determining metal presence by monitoring its decrease in fluorescence signal with the prevalence of any APC masking its effects on metal‐binding induced fluorescence.

## Materials and methods

### Chemicals and reagents

Ammonium acetate, methanol, potassium phosphate monobasic and potassium phosphate dibasic were purchased from Fisher Scientific (Loughborough, UK). Purified APC (A7472, #SLBZ5120), copper acetate, silver carbonate, iron acetate, zinc acetate and nitrilotriacetic acid (NTA) were purchased from Sigma‐Aldrich (St. Louis, CA, USA). Volatile salts (acetate and carbonate) of these metals were used to minimise non‐specific binding and interference from non‐volatile salts (e.g., sulfate and nitrate). Metal ion stocks were prepared either in ultrapure water or in 100 mm ammonium acetate and stored at 4 °C with serial dilution performed when needed. Fe^2+^ can readily oxidise to Fe^3+^ in solution, thus, for clarity we have labelled it as Fe^2+/3+^ throughout. Iron (II) acetate was prepared in water and serial dilution performed into ammonium acetate immediately before mixing with the protein samples. This minimised the ability of iron acetate to form an insoluble aggregate (iron ammonium acetate). For preparation of the chelated iron, nitrilotriacetic acid (free acid) was solubilised in two equivalents of ammonium hydroxide solution to give a 50 mm stock of the ammonium salt, this was then mixed with aqueous iron (II) acetate to give 1 : 1 Fe:NTA solution.

### Algae growth

*Arthrospira* *maxima* (synonym *Limnospira* *maxima*, isolated from Lake Chad, Africa) was supplied as a 50 mL culture by the CCAP (reference 1475/9) and grown in 50 : 50 artificial seawater/blue‐green (ASW : BG) liquid media at 20 °C under a 12 : 12 h light/dark regime in a MicroPharos PBR™ (Xanthella, Oban, UK).

### Algae lysis and phycobiliprotein purification

Fresh *A. maxima* cell pellet was taken and lysed in an equivalent volume of 50 mm potassium phosphate (pH 7.0) using a combination of freeze‐thaw (−80 to +25 °C) cycles and sonication. The lysate was then centrifuged at 16 000 ***g*** for 5 min. The blue‐green supernatant was clarified with a stepwise ammonium sulfate (AmSO_4_) precipitation, collecting the blue supernatant after 25% AmSO_4_ and blue precipitate after 60% AmSO_4_ containing the phycobiliproteins. After each step centrifugation was performed at 16 000 ***g*** for 15 min. The mixture of phycobiliproteins was further purified using size exclusion chromatography (Superdex Increase 200, 10/300 GL; GE Healthcare, Uppsala, Sweden) into 100 mm ammonium acetate (pH 6.8). Fractions corresponding to hexameric phycobiliproteins were pooled. For higher purity PC, the 60% AmSO_4_ precipitate was buffer exchanged into 50 mm ammonium acetate (pH 5.0) and PC separated from APC by anion exchange chromatography (HiTrap DEAE FF; GE Healthcare) with a linear gradient to 50 mm ammonium acetate (pH 3.8) [37]. For all purifications, fractions were further exchanged into 100 mm ammonium acetate (pH 6.8) with an Amicon Ultra 0.5 mL centrifugal concentrator with a 30k MWCO (Merck Millipore, Darmstadt, Germany). All phycobiliproteins were stored at 4 °C in the dark to prevent degradation of the bilins and protein complexes.

### Visible light and fluorescence spectroscopy

Purified APC was diluted to 0.5 μm hexamer (3 μm bilin) in 50 mm potassium phosphate buffer (pH 7.0) in the absence or presence of metal ions (Ag^+^, Cu^2+^, Fe^2+^, Zn^2+^; 60 μm) and incubated for 24 h. Purified PC was diluted to 1.3 μm (12 μm bilin) in 50 mm potassium phosphate buffer (pH 7.0) in the absence or presence of metal ions (Ag^+^, Cu^2+^; 60 μm). Absorbance spectra were acquired on a 7200‐model visible spectrophotometer (Jenway, Stone, UK) over the 400–750 nm range or a Biowave 3 spectrophotometer (Biochrom, Cambridge, UK) over the 250–800 nm range. Fluorescence emission spectra were acquired on a Cary Eclipse fluorometer (Agilent, Santa Clara, CA, USA) over the 645–750 nm range for APC or 600–750 nm range for PC after excitation at 645 nm for APC or 580 nm for PC; 5 nm excitation slit width, 2.5 nm emission slit width, PMT voltage of 675 V, calibrated to deionised water Raman sample. The quenching effect of metal ions is expressed as the relative fluorescence (at 663 nm for APC or 651 nm for PC) of the phycobiliprotein in the presence versus absence of metal ions; five technical replicates were recorded for each condition and the standard deviation between replicates reported.

### Native mass spectrometry

All metal‐binding native MS experiments were performed at bilin‐normalised concentrations; for purified APC, this was 1 μm APC (hexamer with six bilins, 6 μm bilin); for purified PC, this was 2.7 μm PC (hexamer with nine bilins, 24 μm bilin); for *A. maxima* extract, this was approximately 2.5 μm PC and 0.2 μm APC (hexamer to give 24 μm bilin). The concentration of phycobiliproteins was determined using a DS‐11 spectrophotometer (DeNovix, Wilmington, NC, USA) as follows: for purified APC, absorbance at peak maxima 652 nm with a molar extinction coefficient of 700 000 m·cm^−1^; for PC‐containing samples, absorbance maxima at 280, 620 and 652 nm using the equation defined by Bennett and Bogorad [38]. Metals were added to a final concentration of 6–240 μm for Cu^2+^, Fe^2+^ and Zn^2+^ or 3–60 μm for Ag^+^—due to aggregation of silver in the emitter tip, lower relative concentrations were used. Samples were typically incubated for 45 min before measurement with time course measurements performed at intervals over 24 h where stated.

Native MS experiments were performed using nano‐electrospray ionisation. Borosilicate emitter tips (1.2 mm o.d., 0.68 mm i.d.) were pulled in‐house (P‐1000 micropipette puller; Sutter Instruments, Novato, CA, USA) and gold coated (sputter coater, Agar Scientific, Stansted, UK). An Orbitrap Eclipse Tribrid mass spectrometer (Thermo Fisher Scientific, Bremen, Germany) was used for all MS experiments in positive ion mode; calibrated with positive‐ion mode FlexMix (Pierce, Thermo Fisher Scientific). To maximise transmission of high mass ions while minimising protein complex dissociation, the capillary voltage was optimised at 1.0–1.4 kV and ion optics were set as follows: transfer tube was held at 250 °C, in‐source dissociation of 0 V, S‐lens RF of 120%, intact protein and high‐pressure mode. The Orbitrap was used for all mass spectra acquisition, typically with a mass range of 1000–8000 *m*/*z* and resolution of 15 000 (at 400 *m/z*). The normalised automatic gain control (AGC) was set to 100% (400 000), maximum injection time of 50–200 ms and 5–10 μscans.

### Data processing

All mass spectra were processed using Xcalibur 4.1 (Thermo Fisher Scientific). Predicted masses of the APC and PC proteins were calculated from the metagenome sequences for *A. maxima* CCAP 1475/9 (European Nucleotide Archive project number PRJEB45336) with associated post‐translational modifications referenced from UniProt accessions for *Arthrospira* *platensis* (see Table 1), as described previously [39]. For all oligomer states, masses were calculated for up to *n* metal adducts, where *n* is the total number of PCB moieties present (i.e., six and nine for APC and PC hexamer).

**Table 1:** Experimental molecular weight assignments for phycobiliproteins. The theoretical and observed average molecular weights for PC and APC including predicted post‐translational modifications: N‐terminal methionine loss (−131.2 Da, APC α subunit), addition of PCB (+586.7 Da, all subunits) and methylation (Me) of asparagine (+14.0 Da, APC β and PC β). Uniprot references: P72504, P72505, P72508 and P72509.

Protein complex	Modifications	Expected MW (Da)	Observed MW (Da)	Mass deviation
PC				
αβ dimer	+3 × PCB + Me	37467.6	37466.8 ± 0.2	0.002%
α_3_β_3_ hexamer	+9 × PCB + 3× Me	112402.8	112414.1 ± 3.6	0.01%
APC				
αβ dimer	−N‐Met + 2 × PCB + Me	35777.8	35776.7 ± 0.03	0.003%
α_3_β_3_ hexamer	−3 × N‐Met + 6 × PCB + 3 × Me	107333.4	107357.1 ± 1.8	0.02%

## Conflict of interest

The authors declare no conflict of interest.

## Author contributions

ACL conceived the idea for the project together with JBC. JBC and JKS acquired the data. All authors analysed the data. JBC and ACL wrote the paper with input from JKS.

### Peer review

The peer review history for this article is available at https://publons.com/publon/10.1111/febs.16396.

## Data Availability

Supplementary data is openly available from the University of Birmingham data archive at DOI (https://doi.org/10.25500/eData.bham.00000780).
